# Cholesterol-enriched diet causes age-related macular degeneration-like pathology in rabbit retina

**DOI:** 10.1186/1471-2415-11-22

**Published:** 2011-08-18

**Authors:** Bhanu Dasari, Jaya RP Prasanthi, Gurdeep Marwarha, Brij B Singh, Othman Ghribi

**Affiliations:** 1 Department of Pharmacology, Physiology and Therapeutics, University of North Dakota School of Medicine and Health Sciences, Grand Forks, North Dakota, 58202, USA; 2 Department of Biochemistry and Molecular Biology, University of North Dakota School of Medicine and Health Sciences, Grand Forks, North Dakota, 58202, USA

## Abstract

**Background:**

Alzheimer's disease (AD) and age-related macular degeneration (AMD) share several pathological hallmarks including β-amyloid (Aβ) accumulation, oxidative stress, and apoptotic cell death. The causes of AD and AMD are likely multi-factorial with several factors such as diet, environment, and genetic susceptibility participating in the pathogenesis of these diseases. Epidemiological studies correlated high plasma cholesterol levels with high incidence of AD, and feeding rabbits with a diet rich in cholesterol has been shown to induce AD-like pathology in rabbit brain. High intake of cholesterol and saturated fat were also long been suspected to increase the risk for AMD. However, the extent to which cholesterol-enriched diet may also cause AMD-like features in rabbit retinas is not well known.

**Methods:**

Male New Zealand white rabbits were fed normal chow or a 2% cholesterol-enriched diet for 12 weeks. At necropsy, animals were perfused with Dulbecco's phosphate-buffered saline and the eyes were promptly removed. One eye of each animal was used for immunohistochemistry and retina dissected from the other eye was used for Western blot, ELISA assays, spectrophotometry and mass spectrometry analyses.

**Results:**

Increased levels of Aβ, decreased levels of the anti-apoptotic protein Bcl-2, increased levels of the pro-apoptotic Bax and gadd153 proteins, emergence of TUNEL-positive cells, and increased generation of reactive oxygen species were found in retinas from cholesterol-fed compared to normal chow-fed rabbits. Additionally, astrogliosis, drusen-like debris and cholesterol accumulations in retinas from cholesterol-fed rabbits were observed. As several lines of evidence suggest that oxidized cholesterol metabolites (oxysterols) may be the link by which cholesterol contributes to the pathogenesis of AMD, we determined levels of oxysterols and found a dramatic increase in levels of oxysterols in retinas from cholesterol-fed rabbits.

**Conclusions:**

Our results suggest that cholesterol-enriched diets cause retinal degeneration that is relevant to AMD. Furthermore, our data suggests high cholesterol levels and subsequent increase in the cholesterol metabolites as potential culprits to AMD.

## Background

Age-related macular degeneration (AMD) is a retinal degenerative disease that involves photoreceptors, retinal pigment epithelium (RPE), Bruch's membrane, and choriocapillaris. Drusen, extracellular deposits located between the RPE and Bruch's membrane, are a major hallmark of AMD [1,2]. Drusen contains histochemically detectable lipid including cholesterol in unesterified and esterified forms [3]. In addition to drusen deposits, oxidative stress, apoptosis and accumulation of β-amyloid peptide are also hallmarks of AMD [4]. Interestingly, these hallmarks are also characteristics of Alzheimer's disease (AD) [5,6]. Furthermore, Aβ accumulation is the leading neuropathological change that correlates with the diagnosis of AD, and is considered a key player in the pathogenesis of AD by inducing oxidative stress and apoptotic cell death. Aβ levels are regulated by generation from amyloid precursor protein (APP) upon initial cleavage by Beta-secretase 1 (BACE-1) and degradation by enzymes that include insulin-degrading enzyme (IDE). Aβ accumulation has also been shown to be associated with drusen in eyes from AMD patients [7-9] as well as mice models for AMD [10] and Aβ immunization has been considered a pertinent therapeutic approach for both AD and AMD [11].

There is increasing evidence of a link between AD and retinal diseases including glaucoma and AMD, as evidenced with the deposition of Aβ peptide in both diseases (see review [12]). Visual problems have been observed during the initial stages of AD [13]. Reduction in the number of ganglion cells and in the thickness of the nerve-fiber layer has been observed in AD patients [14]. The causes of AMD and AD are not well defined, but several factors including diet, environment, and genetic susceptibility likely contribute to the pathogenesis of these diseases [15]. Epidemiological and animal studies have suggested a link between high plasma cholesterol levels and AD [16]. As well, high intake of cholesterol and saturated fat have long been suspected to increase the risk for AMD [17]. Cholesterol (free and esterified) has been shown to be highly distributed in the human drusen [18-20]. Drusen may raise the risk of developing AMD [21]. The source of the cholesterol that accumulates in the retina is suggested to derive from both local cells and plasma origins [22-24]. Currently, the mechanisms by which cholesterol may increase the incidence of AMD are not clear. Several lines of evidence suggest that oxidized cholesterol metabolites (oxysterols) may be the link by which cholesterol contributes to the pathogenesis of AMD. The oxysterol pathway has been proposed as a unifying hypothesis for the cause of AMD [25,26]. We have shown that cholesterol-enriched diets increase Aβ levels, oxidative stress and cell death in rabbit brains [27]. We have further presented ample evidence that increased levels of 27-hydroxycholesterol, an oxidized cholesterol metabolite, is a potential link that mediates cholesterol-induced AD pathology [28,29]. We have also recently demonstrated that incubation of retinal pigment epithelial cells with the oxysterol 27-hydroxycholesterol caused Aβ accumulation, oxidative stress and apoptosis [30]. Our data suggest that increased oxysterol levels, subsequently to high plasma cholesterol levels, may be a common pathogenic factor for both AD and AMD. However, the extent to which cholesterol-fed rabbit retinas also exhibit increased Aβ levels, oxidative stress and cell death is not well known. As well, the extent to which these features are associated with increased levels of oxysterols in rabbit retinas has not been determined.

In this study, we fed rabbits with a cholesterol-enriched diet for 12 weeks and found that this regimen is associated with increased levels of Aβ, oxidative damage, apoptotic cell death as well as increased cholesterol and oxysterol levels in retinas. Our results suggest a potential link between cholesterol metabolism and retinal degeneration.

## Methods

### Animals and treatments

Male New Zealand white rabbits (1.5-2 years old, 3-5 kg), housed separately in cages in a room with 12 h dark/light cycle, were randomly assigned to 2 groups (n = 6 each) as follows: group 1, normal chow and group 2, chow supplemented with 2% cholesterol (Harlan Teklad Global Diets, Madison, WI). Animals fed with cholesterol-enriched diet and their matched controls were euthanized 12 weeks later. At necropsy, animals were perfused with Dulbecco's phosphate-buffered saline (GIBCO) and the eyes were promptly enucleated. One eye of each animal was used for biochemical analysis and the other was immediately placed in a fixative solution for paraffin embedding. All animal procedures were carried out in accordance with the U.S. Public Health Service Policy on Humane Care and Use of Laboratory Animals and were approved by the Institutional Animal Care and Use Committee at the University of North Dakota.

### Immunohistochemistry

After deparaffinization and rehydration, paraffin sections (7 μm) were washed with PBS, incubated with trypsin enzymatic antigen retrieval solution (Abcam, Cambridge, MA) then blocked with 10% normal goat serum solution for 1 h. This was followed by an overnight incubation at 4°C with mouse monoclonal antibody to 4G8 that detects Aβ (1:100, Signet Laboratories, Dedham, MA), rat monoclonal to GFAP (1:50, Abcam), mouse monoclonal antibody to vitronectin (1:200, Santa Cruz Biotechnology, Santa Cruz, CA) and mouse polyclonal antibody to CYP27A1, the enzyme that converts cholesterol to 27-hydroxycholesterol (1:200, Novus Biologicals, Littleton, CO). Anti-mouse IgG conjugated with Alexa Fluor 488 Dye (Molecular Probes, Eugene, OR) at 1:500 and anti-rat IgG conjugated with Alexa Fluor 594 Dye (Molecular Probes) at 1:500 were used as secondary antibodies and incubated in PBS for 1 h at room temperature. DAPI or propidium iodide was used as a counter stain for visualizing nuclei. Hematoxylin and eosin (H&E) staining was carried out after deparaffinization and rehydration of retinal sections with xylene, ethanol and deionized H_2_O.

### Quantification of Aβ levels by ELISA

Aβ levels (Aβ40 and Aβ42) were quantified in the retina of control and cholesterol-fed rabbits with an ELISA kit (Invitrogen, Carlsband, CA) according to the manufacturer's protocol. Briefly, to measure the amount of Aβ40 and Aβ42, the wet mass of the retina was homogenized thoroughly with 8 × mass of cold 5 M guanidine HCl/50 mM Tris-HCl. The homogenates were mixed for 3-4 h at room temperature and samples were diluted with cold reaction buffer (Dulbecco's phosphate-buffered saline with 5% BSA and 0.03% Tween-20 supplemented with 1 × protease inhibitor cocktail) and centrifuged at 16,000 × *g *for 20 min at 4°C. The supernatant was decanted, stored on ice until use, diluted 1:2 with standard diluent buffer, and quantified by colorimetric sandwich ELISA kits. The quantity of Aβ in each sample was measured in duplicates. Protein concentrations of all samples were determined by standard BCA assay (Pierce Chemical Co, Rockford, IL). Aβ levels were normalized to total protein content in the samples.

### Western blot analysis

Retinas dissected from control and cholesterol-fed rabbit eyes were homogenized in T-PER(tissue protein extraction reagent, Thermo Scientific, Rockford, IL) supplemented with protease and phosphatase inhibitors (Thermo Scientific). Protein concentrations were determined with BCA protein assay reagent (Pierce Chemical Co). Proteins (10 μg) were separated on SDS-PAGE gels followed by transfer to a polyvinylidene difluoride membrane (Biorad, Herculus, CA) and incubated with antibodies to BACE-1 (Mouse, 1:1000, Millipore), IDE (Rabbit, 1:5000, Millipore), HO-1 (Mouse, 1:200, Assay Designs, Ann Arbor, MI), GFAP (Rat, 1:200, Abcam), Bcl-2 (Mouse, 1:100, Santa Cruz Biotechnology), Bax (Mouse, 1:100, Santa Cruz Biotechnology), gadd153 (Mouse, 1:1000, Abcam), CYP27A1 (Rabbit, 1:800, Protein Tech Group, Chicago, IL), and ABCA1 (Mouse, 1:100, Neuromics, Edina, MN). β-actin was used as a gel loading control (Mouse, 1:5000, Santa Cruz Biotechnology). For antibodies of mouse origin, goat anti-mouse secondary antibody conjugated with horseradish peroxidase (HRP, 1:5000, Biorad), for antibodies of rabbit origin goat anti-rabbit secondary antibody (1:5000, Biorad), and for antibodies of rat origin, goat anti-rat secondary (1:500, Santa Cruz Biotechnology) were used. The blots were developed with enhanced chemiluminescence (Immun-star HRP chemiluminescent kit, Bio-Rad). Bands were visualized on a PVDF membrane by using Ultra Violet Products (UVP) Bioimaging System (UVP, Upland, CA) and analyzed by LabWorks 4.5 software. Quantification of results was performed by densitometry and the results analyzed as total integrated densitometric values (arbitrary units).

### TUNEL Assay

The DeadEnd Fluorometric TUNEL assay (Promega, Madison, WI) was performed for detection of apoptosis. The TUNEL staining was performed according to manufacturer's instructions. Briefly, sections were deparaffinized, rehydrated and washed with PBS. Sections were permeabilized with Triton-X, washed with PBS, incubated with terminal deoxynucleotidyl transferase, fluorescein-12-dUTP. The fluorescein-12-dUTP labeled DNA was then visualized directly by fluorescence microscopy. Propidium iodide was used as counter stain for staining nucleus.

### Reactive Oxygen Species measurements

Reactive Oxygen Species (ROS) generation was measured in retinal tissue homogenates using a 2'-7'-dichlorofluorescin-diacetate (DCFH-DA) as well as fluorometric detection of H_2_O_2_. DCFH-DA, a non-fluorescent dye, is cleaved by esterase activity to yield DCFH, which is subsequently oxidized by a variety of ROS to form dichlorofluorescein (DCF), which is fluorescent. Retinas were homogenized in T-PER using a glass homogenizer. Samples containing 25 μg proteins diluted in PBS were incubated with 5 *μ*M DCFH-DA (Sigma) in the dark for 15 min. Fluorescence was measured every 15 min for 1 h with excitation and emission wavelengths of 488 nm and 525 nm, respectively, using a SpectraMax Gemini EM microplate reader (Molecular Devices, Sunnyvale, CA, USA). Values are expressed as relative fluorescence units (RFU). For the measurement of hydrogen peroxide (H_2_O_2_) and peroxidase activity in the retinal samples, we used Amplex Red Hydrogen Peroxide/Peroxidase Assay according to the manufacturer's instructions (Invitrogen). In the presence of peroxidase, the Amplex Red reagent reacts with H_2_O_2 _in a 1:1 stoichiometry and produces the red-fluorescent oxidation product, resorufin. Retinal homogenates of control and cholesterol-fed rabbits were diluted in reaction buffer and added into 96 well plates. For each well, 50 μL of working solution of 100 μM Amplex Red reagent and 0.2 U/mL HRP was added and fluorescence was measured after incubation. For H_2_O_2 _Assay, a standard curve was generated from 0 μM to 5 μM and H_2_O_2 _concentrations of retina samples were deduced from the standard curve. Similarly, for peroxidase activity determination, 100 μM Amplex Red reagent containing 2 mM H_2_O_2 _was added to retinal homogenates and after incubation fluorescence was measured. Peroxidase standard curve was generated in the range from 0 to 2 mU/ml. Peroxidase activity of retinal samples were deduced from standard curve. Resorufin fluorescence was measured using a SpectraMax Gemini-EM (Molecular Devices) with excitation at 530-560 nm and emission at 590 nm.

### Total cholesterol quantification

Total cholesterol levels in the control and cholesterol-fed rabbit retina samples were quantified by colorimetric detection using cholesterol/cholesteryl ester quantification kit (BioVision Research Products, Mountain View, CA) as per the manufacturer's instructions. Cholesterol was extracted from the retina samples in a solution containing a mixture of chloroform: isopropanol: NP-40 (7:11:0.1). The extract was centrifuged at 15,000 × g and the organic phase was transferred to a new tube. The organic phase liquid was air dried at 50°C to remove chloroform and subjected to vacuum for 30 min to remove trace organic solvents. The dried lipids were dissolved in 200 μL of cholesterol assay buffer provided with the kit until the samples were homogeneous by either sonicating or vortexing. Standards were prepared as per the manufacturer's instructions. 1 μL of the extracted sample adjusted to 50 μL/well with cholesterol assay buffer was used per assay. 50 μL of the reaction mix (containing 44 μL of cholesterol assay buffer, 2 μL of cholesterol probe, 2 μL of cholesterol enzyme mix and 2 μL of cholesterol esterase all provided in the kit) was added to each well containing standards and samples. After 1 h reaction at 37°C the absorbance was read at 570 nm. The concentration of cholesterol in each sample was calculated using the standard curve and expressed as mg/g tissue.

### Oxysterol levels measurement

Oxysterols were quantified in retinas from control (n = 3) and cholesterol-fed (n = 3) rabbits using a 4000 QTRAP liquid chromatography mass spectrometer (Applied Biosystems) as previously described [31].

### Statistical analysis

GraphPad Prism software 4.01 was used for statistical analysis. Quantitative data are presented as mean values ± SEM. The significance of differences between the control and cholesterol-treated group was assessed by unpaired Student's *t *test, with *P *< 0.05 considered statistically significant.

## Results

### Aβ levels were increased in retinas of cholesterol-fed rabbits

**Figure 1:**
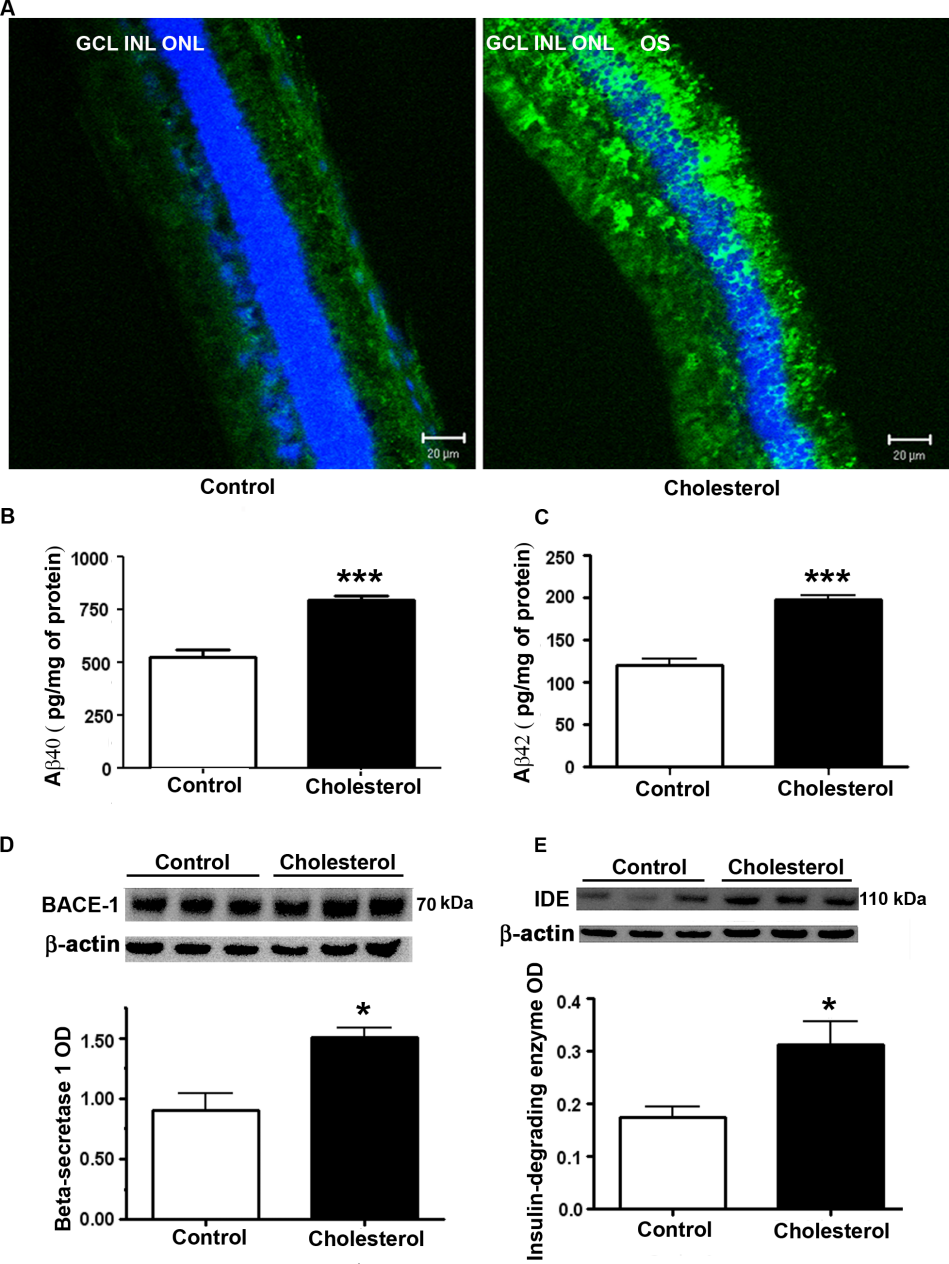
**4G8 immunoreactivity and Aβ levels were increased in cholesterol-fed rabbit retina**. **A**. Immunohistochemical analysis of retinal sections with 4G8 (green), an antibody that detects Aβ, showed increased immunoreactivity in the cholesterol-fed rabbits compared to control. DAPI (blue) is a nuclear counter stain. Bar = 20 μm. OS, ONL, INL, GCL are outer segments, outer nuclear layer, inner nuclear layer and ganglion cell layers respectively. **B, C**. Aβ quantification by ELISA showed an increase in levels of Aβ40 and 42 in the retinal samples of cholesterol-fed rabbits compared to normal rabbits. **D**. BACE-1 and **E**. IDE levels are increased in retinas from cholesterol-fed rabbits. *p < 0.05, ***p < 0.001 vs control.

Immunohistochemical analysis with laser scanning confocal microscopy showed increased immunoreactivity to Aβ peptide in retinas from the cholesterol-fed rabbits compared to control rabbit as determined with 4G8 antibody (Figure 1A). Aβ monoclonal antibody 4G8 is reactive to amino acid residues 17-24 of Aβ and also reacts with APP. The increase in Aβ staining is detectable in the photoreceptor outer segments (OS), outer nuclear layer (ONL), inner nuclear layer (INL) and also in the ganglion cell layers (GCL). Aβ quantitation by ELISA also showed an increase in both Aβ40 and Aβ42 forms in the retinal samples of cholesterol-fed rabbits compared to normal chow-fed rabbits (Figure 1B, C). Aβ levels are regulated by generation from APP upon initial cleavage by BACE-1 and degradation by IDE. Western blot analyses demonstrate that BACE-1 and IDE levels were increased in cholesterol-fed rabbit retinas (Figure 1D, E). These results suggest that both formation and degradation of Aβ are enhanced by the cholesterol-enriched diet and that the increase in generation by BACE-1 exceeds the degradation rate of Aβ peptide by IDE.

### Cholesterol-fed rabbit retinas show increased oxidative stress

**Figure 2:**
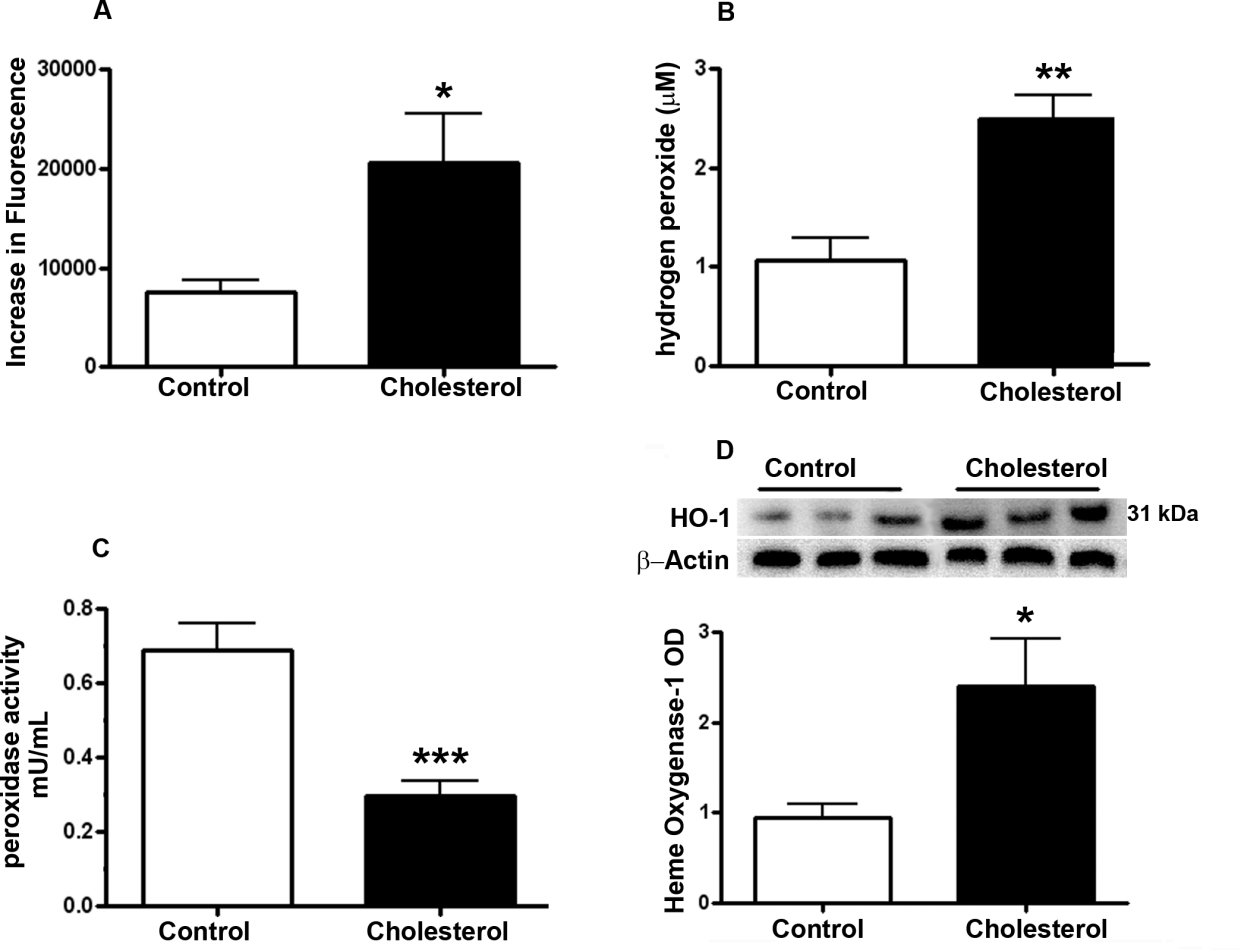
**Retinas of cholesterol-fed animal exhibit an increase in oxidative stress**. **A**. The cholesterol-enriched diet significantly increased ROS levels in the retinas as demonstrated by DCFH-DA. **B, C**. Fluorimetric detection of H_2_O_2 _and peroxidase activity by Amplex Red Assay showed an increase in H_2_O_2 _levels in cholesterol-fed rabbit retinas and decreased peroxidase activity. **D**. HO-1 levels were increased in the retinas of cholesterol-fed rabbits as shown by Western blotting. *p < 0.05;**p < 0.01; ***p < 0.001 vs control.

The nonfluorescent dichlorofluorescein (DCFH), upon oxidation is converted to DCF and emits fluorescence. Because DCFH can be oxidized by various ROS, the increase of DCF fluorescence therefore reflects an overall oxygen species index in cells. The cholesterol-enriched diet significantly increased ROS levels in retinas compared to control animals fed normal diet as demonstrated by DCFH-DA (Figure 2A). Fluorimetric detection of H_2_O_2 _and peroxidase by Amplex Red Assay showed an increase in H_2_O_2 _levels and a decrease in peroxidase activity in retinas from cholesterol-fed rabbits compared to retinas from control rabbits (Figure 2B, C).

Levels of the oxidative stress sensor HO-1 were determined by Western blotting. The levels of HO-1 were significantly higher in retinas of cholesterol-fed compared to normal chow-fed rabbits (Figure 2D). All together, these results show that retinas from cholesterol-fed rabbits were subjected to oxidative stress.

### Cholesterol- enriched diet caused retinal morphological changes

**Figure 3:**
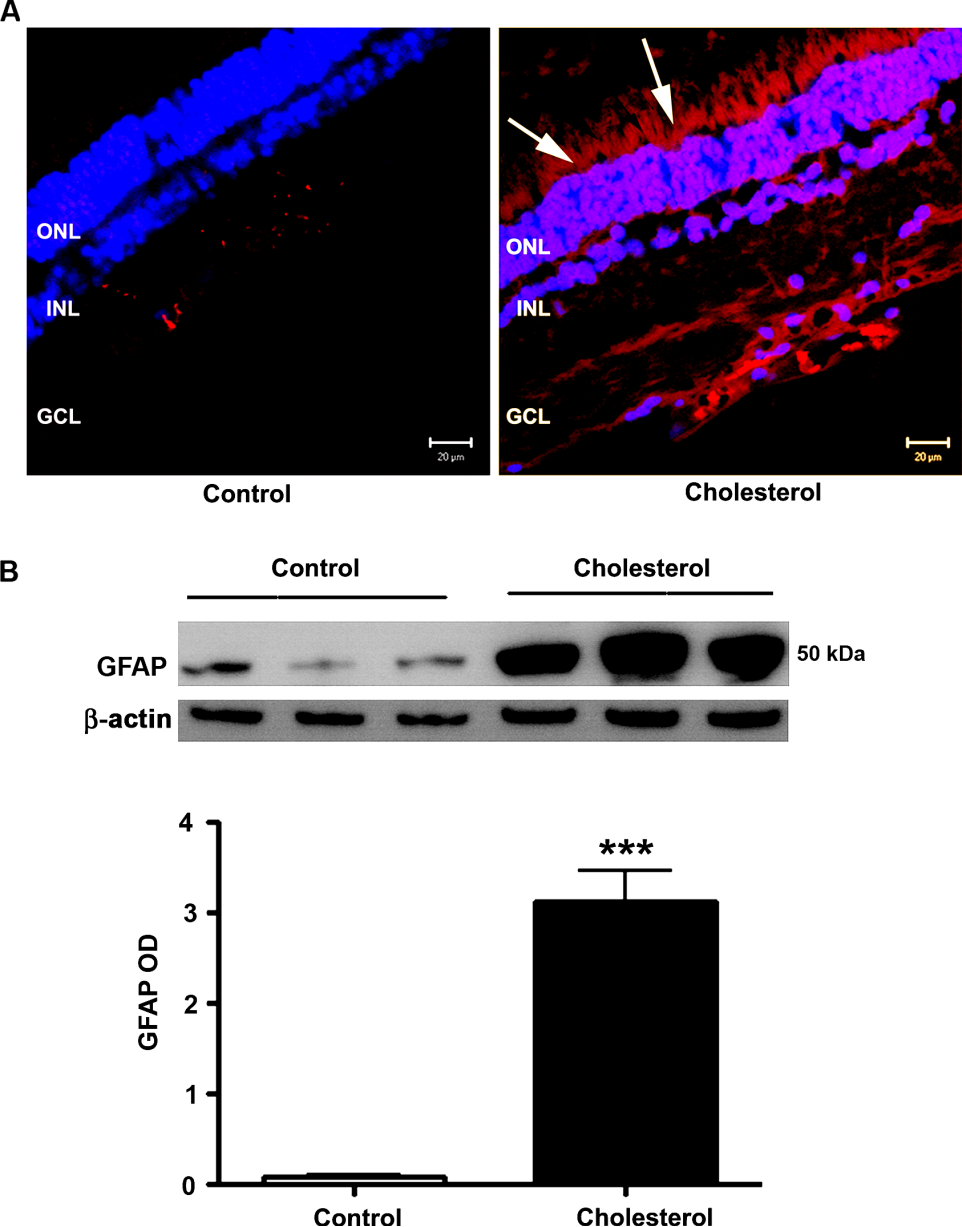
**Cholesterol-enriched diet increased GFAP expression in retina**. **A**. Cholesterol-fed rabbit retinal sections showed an increase in the expression of GFAP (Red). DAPI (blue) is a nuclear counter stain. **B**. Western blot results also showed elevated GFAP levels. ONL, INL, GCL are outer nuclear layer, inner nuclear layer and ganglion cell layers respectively. ***p < 0.001 vs control. Bar = 20 μm.

**Figure 4:**
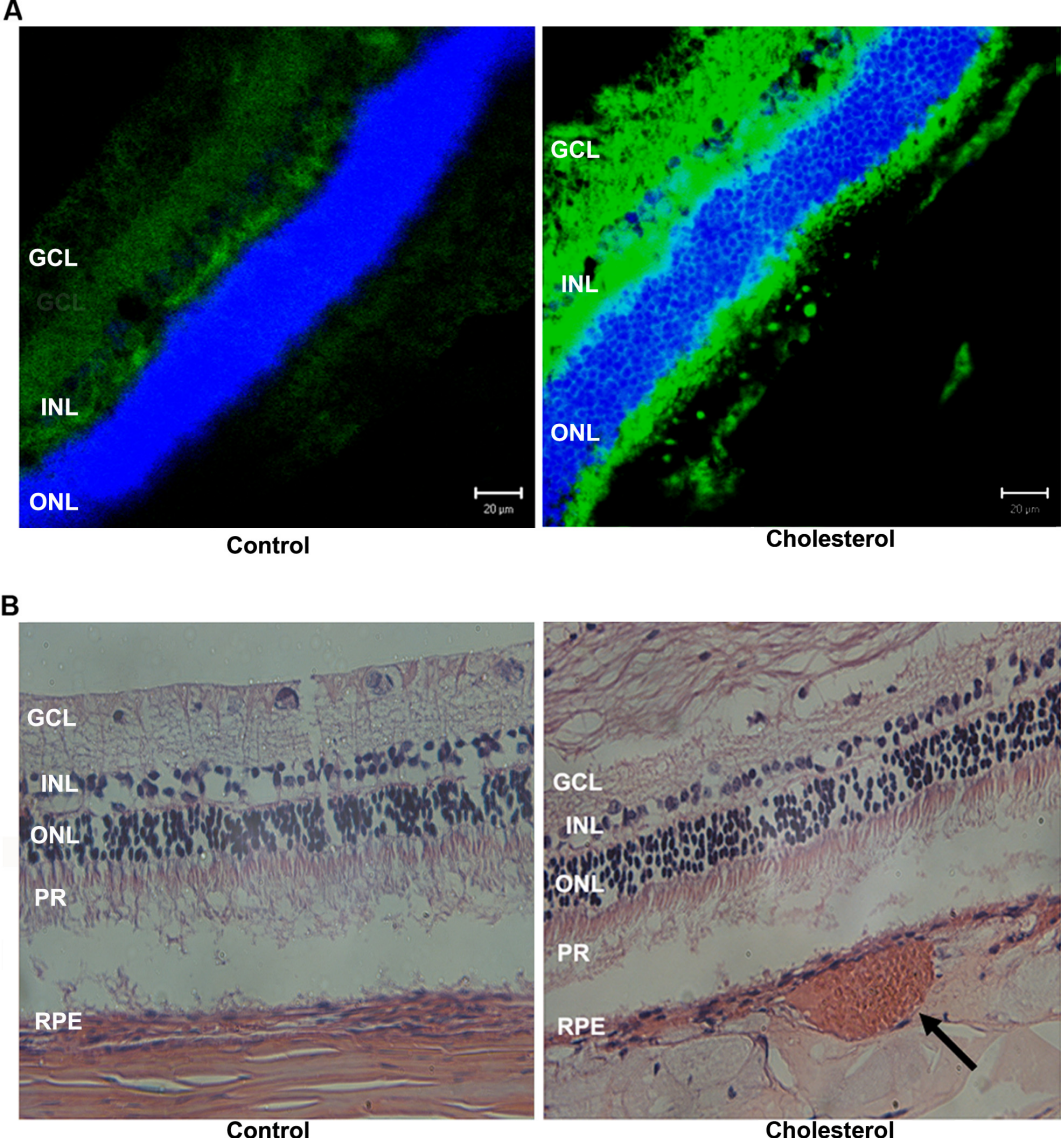
**Cholesterol-enriched diet caused retinal morphological changes**. **A**. Confocal microscopy showed an increase in immunoreactivity of vitronectin antibody (green) in the retinas of rabbits fed with cholesterol-enriched diet compared to rabbits fed with normal chow. **B**. Drusen like debris were detected under the retinal pigment epithelium (arrow) of cholesterol-fed rabbits. GCL-Ganglion Cell Layer; INL-Inner Nuclear Layer; ONL: Outer Nuclear Layer; PR-Photoreceptors; RPE-Retinal Pigment Epithelium. Bar = 20 μm.

Astrogliosis is one of the remarkable characteristics of astrocytes to respond to oxidative stress insults. Confocal microscopy analyses show significant astrogliosis, as detected with increased immunoreactivity to GFAP in retinas from cholesterol-fed rabbits compared to control rabbits (Figure 3A). The increase in the number of astrocytes occurred in the outer nuclear, and the ganglion cell layers. Western blot analyses also show that levels of GFAP protein are dramatically increased in retinas from cholesterol-fed rabbits compared to control rabbits (Figure 3B). Vitronectin, an adhesive glycoprotein, is a common component of extracellular matrices in adult tissues including Bruch's membrane [32] and is expressed in retina. Confocal microscopy showed an increase in immunoreactivity for vitronectin antibody in the retinas of rabbits fed with cholesterol-enriched diet compared to rabbits fed with normal chow (Figure 4A). H&E staining, analyzed by light microscopy, showed necrotic debris suggestive of drusen-like debris in retinas from cholesterol-fed rabbits. These drusen-like debris are localized under the retinal pigment epithelium (Figure 4B).

### Cholesterol- enriched diet caused apoptotic cell death

**Figure 5:**
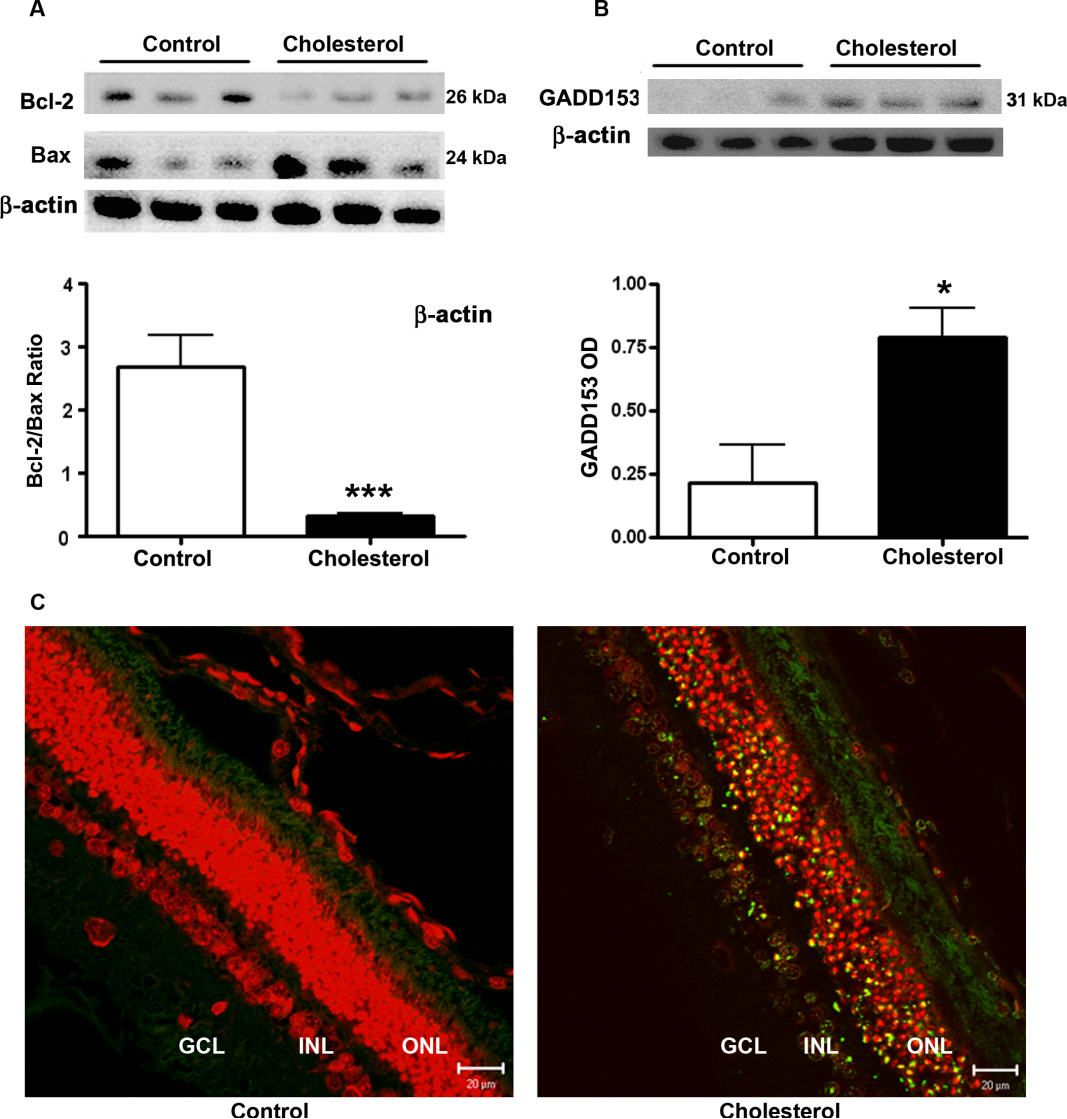
**Cholesterol-enriched diet caused apoptotic cell death**. **A**. Western blot results showed a decrease in the levels of the anti-apoptotic protein Bcl-2 and an increase in levels of the pro-apoptotic protein Bax levels in the retinas of rabbits fed with cholesterol-enriched diet compared to control rabbits. **B**. Levels of the endoplasmic reticulum stress marker gadd153 were increased in cholesterol-fed rabbits as shown by Western blotting. **C**. While no TUNEL-positive cells were detected in retinas from control rabbits, a large number of TUNEL-positive cells were observed in retinas of cholesterol-fed rabbits. Propidium iodide (red) was used as a counterstain for nuclei. Bar = 20 μm. *p < 0.05, ***p < 0.001 vs control.

Western blot results show that levels of the anti-apoptotic protein Bcl-2 were decreased and levels of the pro-apoptotic protein Bax were increased in retinas of cholesterol-fed rabbits in comparison to levels of these proteins in retinas of control rabbits (Figure 5A). In addition to Bcl-2 and Bax, levels of gadd 153 were increased in cholesterol-fed rabbits (Figure 5B). Gadd 153 (also called CHOP), a transcription factor that is activated by stress to the endoplasmic reticulum, triggers cell death by mechanisms that may include generation of ROS, downregulation of Bcl-2, and upregulation of Bax [33]. These results indicate that stress to the endoplasmic reticulum is involved in the deleterious effects of cholesterol-enriched diet in retinas. TUNEL assay detects the fragmented DNA of apoptotic cells by catalytically incorporating fluorescein-12-dUTP at 3'-OH DNA ends using the Terminal Deoxynucleotidyl Transferase, and apoptotic cells fluoresce green color. TUNEL staining showed no apoptotic cells in the control retinas whereas an extensive staining was observed in the retinas of cholesterol-fed animals (Figure 5C). Propidium iodide was used as nuclear counter stain. Yellow color is the merge of green color which indicates fragmented DNA and red color that indicates nucleus.

### Cholesterol- enriched diet disturbed cholesterol homeostasis in the retina

The extent to which cholesterol-enriched diet increases the accumulation of cholesterol and major oxysterols were determined in rabbit retinas (Table 1). Total cholesterol levels were increased in cholesterol fed rabbit retinas compared to control retina. The amount of cholesterol in control retinas was 0.1024 ± 0.049 mg/g of tissue, whereas in cholesterol-fed rabbit retina the concentration of cholesterol increased to 0.8427 ± 0.003 mg/g of tissue. Cholesterol is also eliminated through enzymatic conversion to polar oxysterols that include 27**-**hydroxycholesterol, 24**-**hydroxycholesterol, and 22**-**hydroxycholesterol by CYP27A1, CYP46A1, and CYP11A1 respectively. All these enzymes were found to be expressed in the retina [34]. Mass spectrometry analysis showed a marked increase in the levels of oxysterols 27**-**hydroxycholesterol, 24**-**hydroxycholesterol, and 22**-**hydroxycholesterol in cholesterol-fed rabbit retinas (Table 1). We also measured the other enzymatically-generated oxysterols, 7α-hydroxycholesterol, 4β-hydroxycholesterol and 25-hydroxycholesterol, and found an increase in their levels in retinas from cholesterol-fed rabbits.

**Table 1:** Total cholesterol and oxysterol levels in control (n = 3) and cholesterol-fed rabbit retina (n = 3)

	Control retina	Cholesterol-fed retina
**Total cholesterol** **mg/g tissue**	0.1024 ± 0.028	0.8427 ± 0.002 ***

**4β-hydroxycholesterol** **ng/g tissue**	2491 ± 236	9046 ± 1015**

**7α-hydroxycholesterol** **ng/g tissue**	344.1 ± 49.51	14378 ± 1504 **

**22-hydroxycholesterol** **ng/g tissue**	7.893 ± 1.59	105.6 ± 24.71 *

**24-hydroxycholesterol** **ng/g tissue**	405.3 ± 173.9	1815.32 ± 313.5 *

**25-hydroxycholesterol** **ng/g tissue**	24.39 ± 0.20	835.5 ± 29.77***

**27-hydroxycholesterol** **ng/g tissue**	29.38 ± 8.4	697.2 ± 161.4 *

Values are expressed as mean ± SEM. * p < 0.05; ** p < 0.01; *** p < 0.001 vs control.

**Figure 6:**
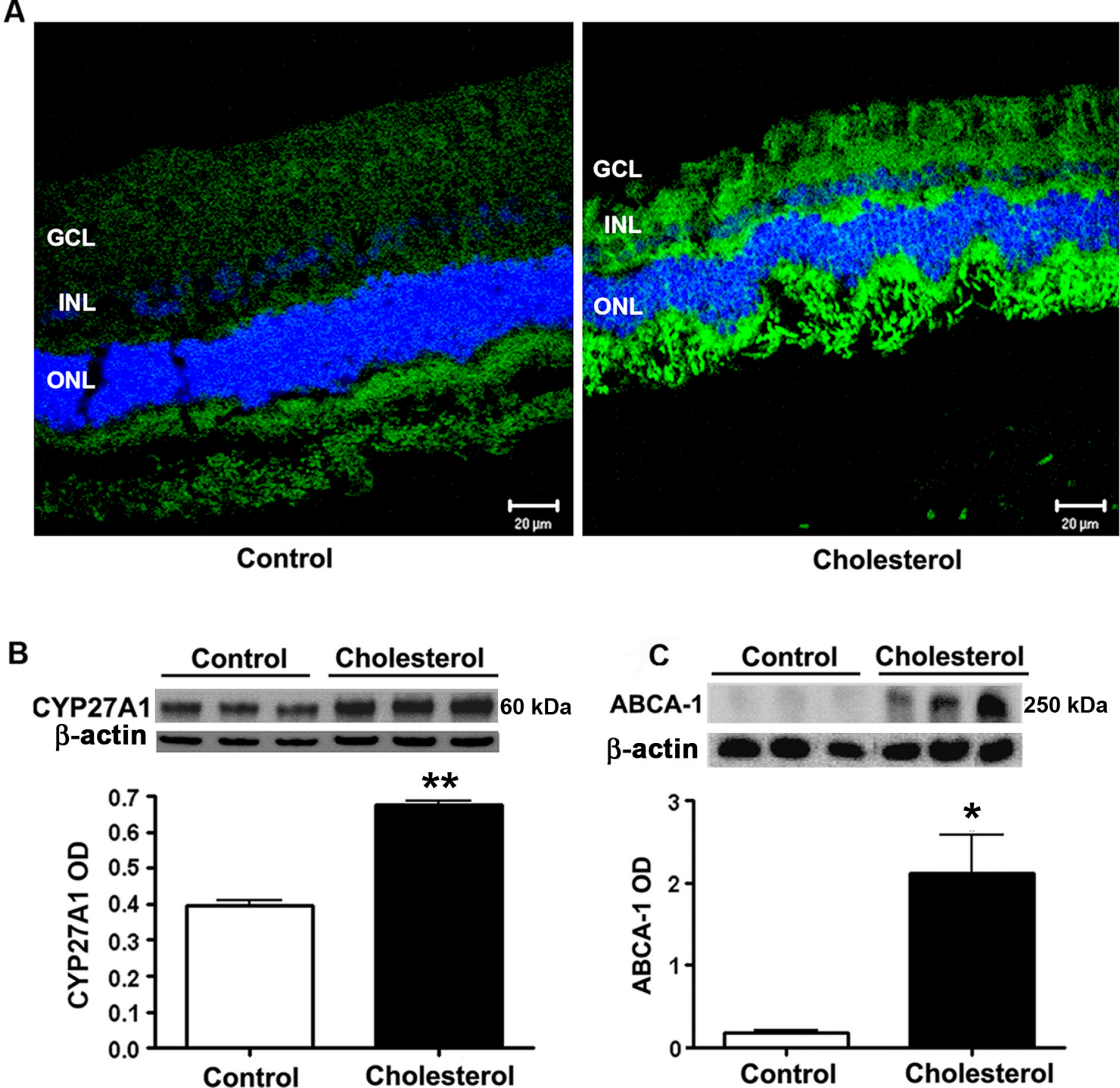
**Cholesterol-enriched diet disturbed cholesterol homeostasis in the retina**. **A**. Cholesterol-fed rabbit retinal sections showed an increase in the expression of CYP27A1 (green). DAPI (blue) is a nuclear counter stain. ONL, INL, GCL are outer nuclear layer, inner nuclear layer and ganglion cell layers respectively. B. Western blotting further confirmed an increase in the expression of CYP27A1. **C**. Western blotting results showed that ABCA-1 levels were increased in the retinas of cholesterol-fed rabbits for facilitating excess cholesterol efflux. *p < 0.05, **p < 0.01 vs control. Bar = 20 μm.

As we have previously shown that 27**-**hydroxycholesterol is toxic to RPE cells [30], we determined levels of CYP27A1, the enzyme that converts cholesterol to 27**-**hydroxycholesterol. CYP27A1 immunoreactivity was dramatically increased in retinas from cholesterol-fed rabbits compared to control rabbits (Figure 6A). Western blotting also showed a significant increase in CYP27A1 levels in retinas from cholesterol-fed rabbits compared to those in retinas of control rabbits (Figure 6B). The cholesterol transporter ATP-binding cassette sub-family A member 1 (ABCA-1) shuttles cholesterol between various cells. Levels of ABCA-1 were dramatically increased in retinas of cholesterol-fed rabbits in comparison to levels in retinas of control rabbits (Figure 6C). All together, these results demonstrate accumulation of cholesterol and increased conversion to oxysterols in retinas as a result of cholesterol-enriched diet.

## Discussion

Strong evidence linking AD and retinal degeneration [12] is the reason behind this study. Retina is an extension of the brain, and has the potential to reflect AD brain pathology. Extracellular Aβ deposition, oxidative stress, and inflammation are implicated in both AD and AMD [4,5]. Ganglion cell death has been recently documented in retinas of AD mouse models [35]. AMD is characterized by the abnormal, extracellular deposits of cellular debris called drusen that are located between Bruch's membrane (BM) and the RPE. Drusen contain various components including amyloid-β peptides [7,36]. Amyloid misfolding-inducing inflammation has been suggested to mediate retinal neurodegeneration [37]. Cholesterol dyshomeostasis has been implicated in the pathogenesis of AD [38] and high-fat diet rich in saturated fatty acids and cholesterol is suggested to be associated with AMD [17]. We determined here the extent to which the cholesterol-fed rabbit model for AD also exhibits retinal pathology relevant to AMD. We demonstrate that cholesterol-enriched diet increased Aβ levels and oxidative stress, induces apoptotic cell death and morphological changes, and alters cholesterol metabolism in rabbit retinas.

Aβ steady state levels are determined by the balance between its production and clearance. Aβ is generated from APP and degraded by several enzymes. Initial cleavage of APP involves BACE-1 enzyme to yield Aβ (Aβ40 and Aβ42). Aβ40 and Aβ42 are then degraded by various enzymes including IDE. We demonstrate here that feeding rabbits a diet rich in cholesterol increased Aβ peptide levels as well as levels of BACE-1. These results suggest that increased Aβ levels in rabbit retinas are derived from an increased production from APP by BACE-1. It may also be possible that increased Aβ transport from the circulation to retinas contributes to the elevated levels of Aβ peptide in retinas. In AD brains, while some studies showed an increase in IDE [39-42], others have showed reduction of IDE in AD [43,44]. In one of the studies, it was suggested that the reduction in IDE activity is not the primary cause of Aβ accumulation but rather is a late stage phenomenon secondary to neurodegeneration [39]. We found here that retinal IDE levels were increased in the cholesterol-fed animals. The increase in IDE levels may have been a mechanism for coping up with the Aβ overload.

As Aβ is a neurotoxic peptide and its accumulation in the retina may promote oxidative damage and cell death, we determined the extent to which accumulation of Aβ peptide is associated with increased ROS and apoptotic cell death. We demonstrate that cholesterol-enriched diet-induced Aβ accumulation is associated with increased oxidative stress. Proteins, carbohydrates, membrane lipids and nucleic acids are all vulnerable to reactive oxygen species damage, and this damage is believed to contribute to the pathogenesis of many diseases. Oxidative injury to cells is associated with several diseases, including AD [45] and AMD [46]. DCFH-DA assay indicated that there is an increase in ROS in the retinas of cholesterol-fed rabbits. Hydrogen peroxide, which is also considered as reactive oxygen species, was increased and peroxidase activity is decreased in cholesterol-fed rabbit retinas. HO-1 is a sensor of oxidative stress that degrades heme, leading to formation of biliverdin and carbon monoxide [47,48]. HO-1 has been shown to be increased in RPE of AMD-affected macula [49]. HO-1 induction is suggested to be an early event in the pathogenesis of sporadic AD [50] and has been demonstrated to be closely associated with neurofibrillary pathology in AD [51]. Neurons of the AD temporal cortex and hippocampus has significantly more HO-1 immunoreactivity than corresponding tissues derived from non-demented controls [52]. We have previously shown that cholesterol-fed rabbits exhibit increased levels of ROS and HO-1 in addition to increased Aβ in the brain [53]. Here we show that HO-1 levels are increased in the retinas of cholesterol-fed rabbits.

Pathogenesis of many retinal and ocular diseases involves apoptosis. Histopathological studies suggest that the retinal pigment epithelium damage occurs first, followed by death of photoreceptors [54], with rod photoreceptor cell loss preceding that of cone photoreceptor cells [55,56]. Photoreceptors underwent apoptosis in 4 of 16 eyes with AMD [57]. It was shown that apoptosis is the cause of photoreceptor cell death in three mouse models of Retinitis pigmentosa [58]. It was also shown that photic exposure triggers apoptosis of photoreceptor cells [59]. Retinal ganglion cells also undergo apoptosis in glaucoma [60,61]. As apoptosis may be involved in AMD and other retinal diseases and cholesterol-enriched diet has been shown to induce apoptosis in rabbit brains [53], we determined the extent to which cholesterol induces apoptotic cell death in retinas. The B-cell lymphoma-2 family of proteins includes both pro- and anti-apoptotic members. Bcl-2 is the most prominent anti-apoptotic member and is an important regulator of photoreceptor cell death in retinal degenerations [62]. Bcl-2 has been shown to decrease apoptosis by facilitating recovery of mitochondrial DNA damage [63]. Our results show that levels of the anti-apoptotic protein Bcl-2 were decreased and levels of the pro-apoptotic protein Bax were increased in retinas of cholesterol-fed rabbits. We also show that cholesterol-enriched diet increased levels of gadd153, a protein that induces cell death and upsets the cellular redox state by depleting cellular glutathione and exaggerating the production of ROS [64].

Development of drusen is one of the earliest clinical features of AMD. Drusen are extracellular deposits of lipids, proteins, glycoproteins, and cellular debris that accumulate between collagenous layer of Bruch's membrane and basal lamina of the retinal pigment epithelium. Recent studies found many drusen constituents including cholesterol, apolipoproteins, and complement components [24,65]. In our study, we detected debris-like material between RPE and choroid capillaries in retinas of cholesterol-fed rabbits. The debris-like material known as drusen is regarded as a hallmark of AMD. In addition, GFAP is strongly upregulated in glial cells in response to neuronal damage and is best known marker for gliosis. Retinal macroglial cells (astrocytes and Müller cells) have been shown to have an active role in normal retinal function [66] as well as in pathology [67]. Upregulation of GFAP expression, an indicator of reactive gliosis, has been demonstrated in response to various retinal pathologies including mechanical injury [68], retinal detachment [69], diabetic retinopathy [70], glaucoma [71], retinal ischemia [72] and photoreceptor degeneration [73]. Increased GFAP expression in macroglia has also been described in retinas with AMD [74-76]. We also found that retinas of cholesterol-fed rabbits exhibit astrogliosis as determined with GFAP immunostaining. Vitronectin has regulatory roles in inflammation and phagocytosis [77]. Increased vitronectin deposition in retina is suggested to participate in the pathogenesis of AMD [32]. Our results also show a marked increase in vitronectin immunoreactivity in retinas from cholesterol-fed rabbits.

All together, our data demonstrate that cholesterol-enriched diets cause structural and morphological changes relevant to AMD. However, the mechanisms by which dietary cholesterol cause these changes in retinas are not well known. Recent genome wide association studies implicated cholesterol metabolism involvement in AMD [78,79]. Cholesterol is constantly taken up by retina via LDL receptors from the circulation in addition to endogenous cholesterol synthesis. Here we found out that cholesterol-enriched diet caused an accumulation of cholesterol in the retina. As excess cholesterol in cells is detrimental, various mechanisms are necessary for cholesterol efflux from cells. These mechanisms include passive diffusion, conversion to oxysterols, and reverse cholesterol transport. ABCA-1 has been shown to play a role in the transport of cholesterol, and was detected in the retina of various organisms [80,81]. ABCA-1 levels are increased in the cholesterol-fed rabbits, implying an increase in the cholesterol content in cells and efflux through ABCA-1. Cholesterol can be oxidized by enzymatic and non-enzymatic pathways. One of the most important enzymatically generated side-chain oxysterol is 27-hydroxycholesterol. This oxysterol is formed from cholesterol by cytochrome P450's CYP27A1 [82]. We found that CYP27A1 expression was increased in retinas of cholesterol-fed rabbits as shown by laser scanning confocal microscopy and Western blotting. We further show an increase in 27-hydroxycholesterol and other oxysterols concentrations in retinas by mass spectrometry. We found that 27-hydroxycholesterol, 22- hydroxycholesterol and 24- hydroxycholesterol levels were increased in cholesterol-fed rabbit retinas providing evidence that elimination of excess cholesterol to oxysterols takes place in the retina. In contrast to our results in rabbit, a recent study could not detect 27-OHC in the bovine and human retinas, but found that its oxidation product, 5-cholestenoic acid is the most abundant oxysterol [83]. We did not measure 5-cholestenoic acid in our study. We also measured other enzymatically generated oxysterols including 7α-hydroxycholesterol, 4β-hydroxycholesterol and 25-hydroxycholesterol catalyzed by CYP7A1, CYP3A4 and cholesterol 25-hydroxylase respectively. These enzymes oxidize cholesterol for various purposes including cholesterol elimination. Even though none of these enzymes were reported to be expressed in the retina, their oxysterol products were found in retinas, most probably coming from blood circulation as retina has access to blood borne lipids [84]. These finding suggest an increase in cholesterol in retinas as well as an increase in cholesterol oxidizing enzyme metabolites. Numerous studies suggested cytotoxic effects of oxysterols are associated with human diseases including AD and AMD (see for review [85]). Increased oxysterol concentrations subsequently to increased cholesterol levels may contribute to the generation of AMD-like pathological hallmarks.

## Conclusions

AMD and AD share many pathological features including accumulation of Aβ, increased oxidative stress, and apoptotic cell death. The causes of AMD and AD are not well known but dietary and environmental factors may contribute to the pathogenesis of these diseases. We demonstrate here that cholesterol-enriched diet induces changes that are relevant to AMD. We further suggest that increased conversion of cholesterol to oxysterols might be a potential mechanism linking high cholesterol to AMD pathology.

## Competing interests

The authors declare that they have no competing interests.

## Authors' contributions

BD performed most experiments and analyzed the data. ELISA experiments were done by JPRP. GM participated in Western blot experiments and provided input in the experimental design. OG and BS conceived the study and supervised the results and wrote the final draft. All authors read and approved the final manuscript.

## Pre-publication history

The pre-publication history for this paper can be accessed here:

http://www.biomedcentral.com/1471-2415/11/22/prepub
